# Lyme Disease and Severe Hyperbilirubinemia: A Rare Presentation of Lyme Disease

**DOI:** 10.7759/cureus.8363

**Published:** 2020-05-30

**Authors:** Zahoor Ahmed, Asad Ur Rehman, Anum Awais, Abdul Hanan, Sarfaraz Ahmad

**Affiliations:** 1 Internal Medicine, King Edward Medical University, Mayo Hospital, Lahore, PAK; 2 Internal Medicine, Pakistan Kidney and Liver Research Institute, Lahore, PAK; 3 Internal Medicine, Fatima Jinnah Medical University, Sir Ganga Ram Hospital, Lahore, PAK; 4 Infectious Disease, Wayne State University, Detroit, USA; 5 Internal Medicine, Saint James School of Medicine, Chicago, USA

**Keywords:** hyperbilirubinemia, lyme disease, doxycycline

## Abstract

A 39-year-old-man presented to the emergency room with a complaint of febrile jaundice and diffuse arthralgia. The patient had a temperature of 100°F, severe jaundice, and scleral icterus. Laboratory workup showed severe hyperbilirubinemia and elevated serum creatinine, and the rest of the serum chemistry was unremarkable. The ultrasound and computed tomography (CT) of the abdomen was normal. The patient had a recent history of travel to an endemic area for Lyme disease. After an extensive workup, all other possible etiologies had been ruled out, and the patient was started on empirical doxycycline by considering the patient’s recent history of travel. Serum serologic test confirmed Lyme disease. His bilirubin and creatinine improved gradually. His fever subsided in three days, and he was discharged with outpatient follow-up. Although hyperbilirubinemia is rare in Lyme disease, it should be considered as a differential diagnosis in patients with severe jaundice and a recent history of travel.

## Introduction

Lyme disease is a tick-borne disease caused by the spirochete *Borrelia burgdorferi*. The signs and symptoms related to the gastrointestinal tract are usually common in the early stage of Lyme disease. However, hepatic impairment in the form of hyperbilirubinemia is extremely uncommon in Lyme disease. Here we present an uncommon case of Lyme disease presented to the emergency room with hyperbilirubinemia.

## Case presentation

A 39-year-old male with a medical history of diabetes mellitus presented to the emergency room for the evaluation of jaundice. He complained of fever for the last seven days that was associated with nausea, intermittent headache, and dry cough. He also had diffuse arthralgia. The patient took over-the-counter antipyretic and analgesic but did not get any relief. The initial evaluation revealed a temperature of 100°F, blood pressure of 110/70 mmHg, heart rate of 101 beats per minute, respiratory rate of 20 per minute, and oxygen saturation of 99% on room air. The physical examination showed severe jaundice with the icteric sclera. He had diffuse arthralgia with tenderness. Cardiac and chest examination was normal. The abdomen was soft with normal bowel sounds and no organomegaly.

Initial laboratory analysis is shown in Tables 1 and 2. The patient’s platelet count, hemoglobin, and hematocrit were within the normal ranges, except white blood cells. His serum metabolic panel was unremarkable, except for elevated total bilirubin and creatinine.

**Table 1:** Results of hematological examination

Parameter	Admission value	Normal range
White blood cell count, cells/mm^3^	17,000	4,000-11,000
Neutrophil (%)	83.5	55-70
Lymphocytes (%)	10.3	20-40
Eosinophils (%)	0.15	0-10
Basophils (%)	0.22	0.5-1.0
Red blood cell count, million cells/mm^3^	4.4	4.35-5.65
Hemoglobin, g/dL	13.8	14-17
Hematocrit (%)	41.9	41-51
Platelet count, cells/mm^3^	200,000	150,000-350,000

**Table 2:** Comprehensive metabolic panel

Parameter	Admission value	Normal range
Sodium (mmol/L)	138	136-145
Potassium (mmol/L)	3.9	3.5-5.0
Chloride (mmol/L)	100	98-106
CO_2_ (mmol/L)	23	23-38
Urea nitrogen (mg/dL)	13	8-20
Creatinine (mg/dL)	4.00	07-1.2
Blood glucose (mg/dL)	101	70-100 (fasting)
Total protein (mg/dL)	5.8	6.0-7.8
Albumin (mg/dL)	4.0	3.5-5.5
Calcium (mg/dL)	8.6	9.0-10.5
Total bilirubin (mg/dL), direct bilirubin (mg/dL), indirect bilirubin (mg/dL)	10.2, 7.8, 2.2	0.3–1.2
Alkaline phosphatase (mg/dL)	80	36–92
Aspartate aminotransferase (IU/L)	50	8–48
Alanine aminotransferase (IU/L)	43	7–55
Erythrocyte sedimentation rate	39	0-22
Prothrombin time (seconds)	12.1	11-13.5
Partial thromboplastin time (seconds)	31	30-40

Extensive workup for severe jaundice involved serum iron studies, cytomegalovirus (CMV) antibody, and antinuclear antibody (ANA), all of which were negative. Common causes of hyperbilirubinemia such as hepatitis A, B, and, C were negative. His low-density lipoprotein (LDL) was 50 mg/dL, high-density lipoprotein (HDL) was 55 mg/dL, serum cholesterol was 180 mg/dL, and serum triglyceride was 152 mg/dL.

On abdominal ultrasound, the liver, gall bladder, and spleen were normal in size and texture. CT of thorax and abdomen was performed, which revealed diffuse pulmonary and hilar infiltrates in both lung bases. It was suspected that the patient had a possible chest infection, and he was started on intravenous ampicillin-sulbactam. The patient’s kidney function was concerning due to high creatinine, but creatinine improved to 2.0 mg/dL on the third hospital day.

On further investigation, the patient reported that he visited Connecticut, USA, one week ago. He had no history of tick bite. Peripheral smear examination of blood was performed, which revealed no abnormality in the morphology of blood cells. Serologic tests for Lyme disease were performed. He was treated with doxycycline at a dose of 100 mg twice a day on the third hospital day. The radiograph of the joints revealed no abnormality.

**Figure 1:**
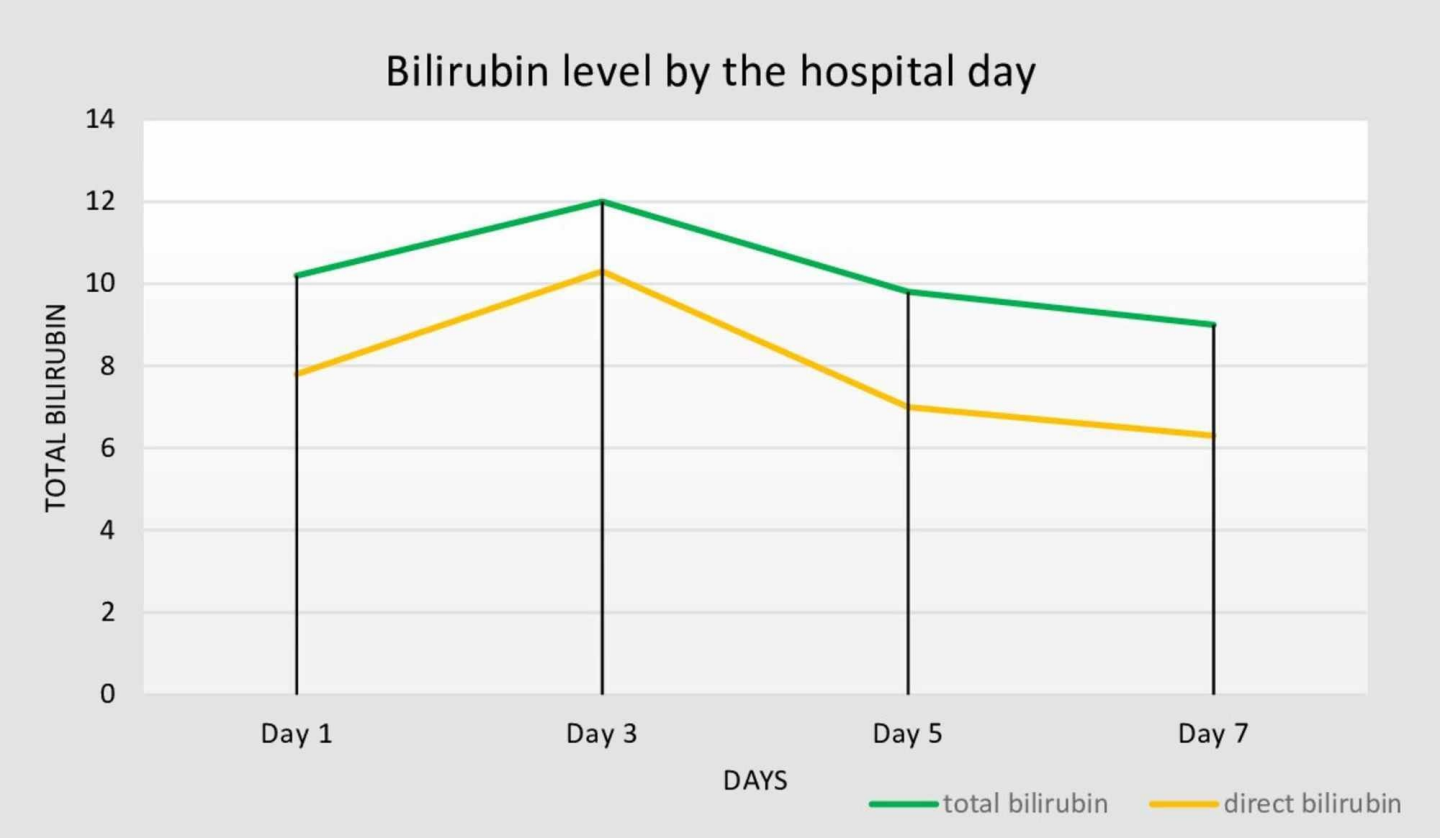
Bilirubin (total and direct) level (mg/dL) by hospital day.

**Figure 2:**
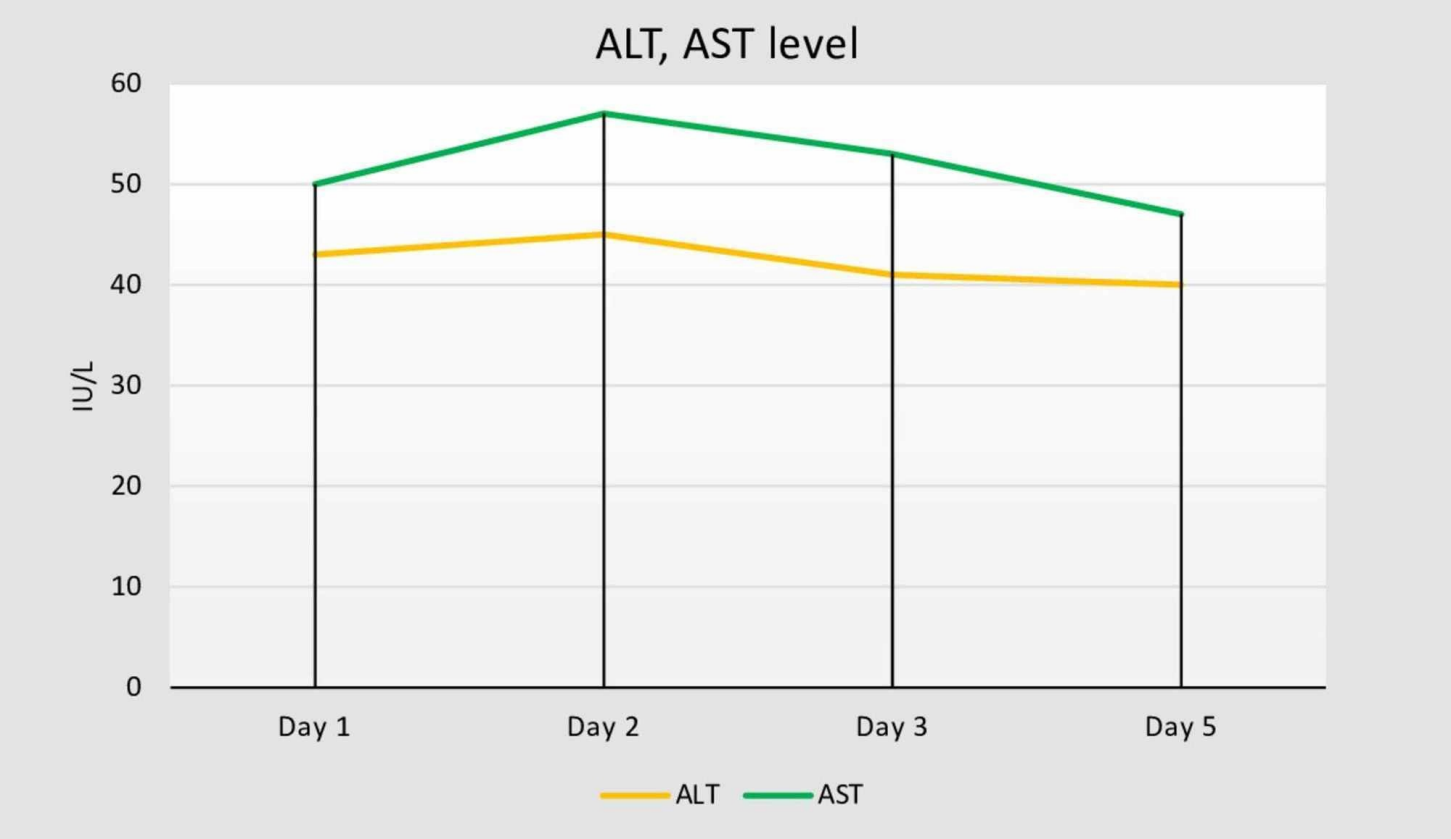
ALT and AST by the hospital day. ALT, alanine aminotransferase; AST, aspartate aminotransferase

His total bilirubin remained high during the hospital stay in the beginning, and his bilirubin was 12.0 mg/dL (direct: 10.3 mg/dL) on the third hospital day (Figure 1). His white blood cell count remained elevated at 18 × 10^3^/µL. However, his liver enzymes remained normal (Figure 2).

The patient’s fever resolved on hospital day 4, and joint pain also got better. Repeat ultrasound of the abdomen was conducted due to the worsening of laboratory results, which revealed no mass or calcification, except few changes in liver echotexture suggesting the possible fatty/fibrotic change. The gallbladder was thin-walled and anechoic, with no stones. Portal vein appeared red-orange, and the hepatic artery was yellow (indicating higher flow velocity). The decision was made for invasive workups such as biopsy and magnetic resonance cholangiopancreatography (MRCP), but the patient denied it.

Serum ELISA (enzyme-linked immunosorbent assay) for Lyme disease was performed. The results of ELISA screen were positive for Lyme disease, which was further confirmed by Western blot. He continued on doxycycline. His bilirubin gradually improved. He was discharged with outpatient follow-up. The patient remained afebrile and symptom-free throughout his hospital stay, without any significant pulmonary or gastrointestinal symptoms. His bilirubin was 9.0 mg/dL on the day of discharge from the hospital.

## Discussion

Abdominal pain, nausea, and vomiting are common gastrointestinal manifestations in the early stages of Lyme disease. Hepatobiliary involvement can also be a sign of early stages of Lyme disease [1]. The number of cases reporting hyperbilirubinemia caused by Lyme disease is few in the literature. Horowitz et al. reported that 40% of patients can have abnormal liver enzymes. Among the patients with Lyme disease, the liver function test showed elevated gamma-glutamyl transpeptidase (GGT) (28%) and alanine aminotransferase (ALT) (27%). However, hyperbilirubinemia was observed only in 3% of the patients in this study [1]. Steere et al. also reported that hyperbilirubinemia was not observed in a single patient out of 314 patients diagnosed with Lyme disease [2].

Although Lyme disease is not so common, diagnoses of Lyme disease should be considered for any patient with severe jaundice, significantly in those patients who are at risk of severe infection and have recently traveled to an endemic area, regardless of the presence of rash. Our patient had a recent history of travel to an endemic area and presented to the hospital with signs and symptoms of fever, joint pain, and jaundice. His high creatinine justifying acute kidney injury could be attributed to Lyme disease associated nephritis [3].

Lyme disease can be diagnosed based on the clinical presentation, recent travel history to an endemic area with a possible record or probable exposure of tick bite, and serologic testing [4,5]. Supporting serum serology evidence is necessary to confirm the diagnosis of Lyme disease. The Centers for Disease Control and Prevention (CDC) endorses a two-tier approach for the detection of *B. burgdorferi* specific immunoglobulin (Ig) M and IgG antibodies [6]. ELISA test has less specificity to detect anti-Borrelia antibodies, and Western blotting, a more specific test, is used to exclude the false-positive ELISA samples [6,7]. The combination of both ELISA and Western blot provides the highest sensitivity and specificity for laboratory diagnosis of Lyme disease. After an extensive workup, all other possible causes of increased bilirubin were ruled out, and his symptoms resolved in two weeks after treatment with doxycycline.

## Conclusions

Severe jaundice could be a presenting sign of Lyme disease, though it is highly uncommon. Lyme disease should be considered as a differential diagnosis for any patient who presents with severe hyperbilirubinemia and has a recent history of travel to an area of endemicity. Hyperbilirubinemia generally resolves in two weeks after starting appropriate treatment.
